# Supplementary material for: Epitope-based chimeric peptide vaccine design against S, M and E proteins of SARS-CoV-2, the etiologic agent of COVID-19 pandemic: an in silico approach
Source: PeerJ. 2020 Jul 27;8:e9572. doi: 10.7717/peerj.9572 (PMC7394063; doi:10.7717/peerj.9572)
Supplement: Table S2 — The epitopes highlighted in green were considered promising vaccine candidates against B-cells of SARS-CoV-2. [file peerj-08-9572-s004.docx]

**Table S2:** Predicted B-cell epitopes in RBD and NTD regions of S glycoprotein, envelop (E) and membrane (M) proteins of the SARS-CoV-2 through BepiPred-2.0 sequential B-Cell epitope predictor. The epitopes highlighted in green color were considered to be promising vaccine candidates against B-cells of SARS-CoV-2.

| **No.** | **Protein** | **Start** | **End** | **Peptide** | **Length** |
| --- | --- | --- | --- | --- | --- |
| 1 | **RBD region** | 341 | 342 | VF | 2 |
| 2 |  | 344 | 349 | ATRFAS | 6 |
| 3 |  | 351 | 363 | YAWNRKRISNCVA | 13 |
| 4 |  | 372 | 378 | ASFSTFK | 7 |
| 5 |  | 382 | 382 | V | 1 |
| 6 |  | 402 | 427 | IRGDEVRQIAPGQTGKIADYNYKLPD | 26 |
| 7 |  | 440 | 485 | NLDSKVGGNYNYLYRLFRKSNLKPFERDISTEIYQAGSTPCNGVEG | 46 |
| 8 |  | 493 | 506 | QSYGFQPTNGVGYQ | 14 |
|  | | | | | |
| 1 | **NTD region** | 72 | 81 | GTNGTKRFDN | 10 |
| 2 |  | 110 | 113 | LDSK | 4 |
| 3 |  | 146 | 155 | HKNNKSWMES | 10 |
| 4 |  | 161 | 162 | SS | 2 |
| 5 |  | 164 | 164 | N | 1 |
| 6 |  | 172 | 191 | SQPFLMDLEGKQGNFKNLRE | 20 |
|  | | | | | |
| 1 | **E protein** | 6 | 9 | SEET | 4 |
| 2 |  | 57 | 71 | YVYSRVKNLNSSRVP | 15 |
|  | | | | | |
| 1 | **M protein** | 5 | 20 | NGTITVEELKKLLEQW | 16 |
| 2 |  | 40 | 41 | AN | 2 |
| 3 |  | 132 | 137 | PLLESE | 6 |
| 4 |  | 161 | 163 | IKD | 3 |
| 5 |  | 180 | 191 | KLGASQRVAGDS | 12 |
| 6 |  | 199 | 218 | YRIGNYKLNTDHSSSSDNIA | 20 |
