# Supplementary material for: Epitope-based chimeric peptide vaccine design against S, M and E proteins of SARS-CoV-2, the etiologic agent of COVID-19 pandemic: an in silico approach
Source: PeerJ. 2020 Jul 27;8:e9572. doi: 10.7717/peerj.9572 (PMC7394063; doi:10.7717/peerj.9572)
Supplement: Table S3 [file peerj-08-9572-s005.docx]

**Table S3:** Predicted B-cell epitopes in RBD and NTD regions of S glycoprotein, envelop (EBE) and membrane (MBE) proteins of the SARS-CoV-2 through Kolaskar and Tongaonkar antigenicity profiling.

| **No.** | **Start** | **End** | **Peptide** | **Average score** |
| --- | --- | --- | --- | --- |
| **RBD** (Average: 1.042 Minimum: 0.907 Maximum: 1.214) | | | | |
| 1 | 333 | 339 | TNLCPFG | 7 |
| 2 | 359 | 371 | SNCVADYSVLYNS | 13 |
| 3 | 376 | 385 | TFKCYGVSPT | 10 |
| 4 | 430 | 435 | TGCVIA | 6 |
| 5 | 488 | 495 | CYFPLQSY | 8 |
| **NTD** (Average: 1.023 Minimum: 0.866 Maximum: 1.213) | | | | |
| 1 | 63 | 16 | TWFHAIHVS | 9 |
| 2 | 81 | 93 | NPVLPFNDGVYFA | 13 |
| 3 | 115 | 121 | QSLLIVN | 7 |
| 4 | 125 | 134 | NVVIKVCEFQ | 10 |
| 5 | 136 | 146 | CNDPFLGVYYH | 11 |
| 6 | 157 | 163 | FRVYSSA | 7 |
| 7 | 168 | 174 | FEYVSQP | 7 |
| **MBE** (Average: 0.980 Minimum: 0.953 Maximum: 1.002) | | | | |
| **EBE** (Average: 1.032 Minimum: 0.947 Maximum: 1.129) | | | | |
