# Supplementary material for: Epitope-based chimeric peptide vaccine design against S, M and E proteins of SARS-CoV-2, the etiologic agent of COVID-19 pandemic: an in silico approach
Source: PeerJ. 2020 Jul 27;8:e9572. doi: 10.7717/peerj.9572 (PMC7394063; doi:10.7717/peerj.9572)
Supplement: Table S4 [file peerj-08-9572-s006.docx]

**Table S4:** Predicted B-cell epitopes in RBD and NTD regions of S glycoprotein of the SARS-CoV-2 through ABCPred-2.0 B-Cell epitope predictor.

| **Rank** | **Sequence** | **Start position** | **Score** |
| --- | --- | --- | --- |
| **RBD domain (Average Score :0.775)** | | | |
| 1 | GSTPCNGVEGFNCYFP | 476 | 0.91 |
| 2 | LQSYGFQPTNGVGYQP | 492 | 0.90 |
| 3 | TEIYQAGSTPCNGVEG | 470 | 0.89 |
| 4 | FERDISTEIYQAGSTP | 464 | 0.86 |
| 5 | EVRQIAPGQTGKIADY | 406 | 0.85 |
| 5 | CFTNVYADSFVIRGDE | 391 | 0.85 |
| 6 | TGKIADYNYKLPDDFT | 415 | 0.84 |
| 7 | FPNITNLCPFGEVFNA | 329 | 0.82 |
| 8 | FASVYAWNRKRISNCV | 347 | 0.81 |
| 9 | NCVADYSVLYNSASFS | 360 | 0.79 |
| 10 | NGVGYQPYRVVVLSFE | 501 | 0.77 |
| 11 | FSTFKCYGVSPTKLND | 374 | 0.76 |
| 12 | SVLYNSASFSTFKCYG | 366 | 0.74 |
| 13 | EGFNCYFPLQSYGFQP | 484 | 0.73 |
| 14 | YKLPDDFTGCVIAWNS | 423 | 0.71 |
| 14 | VGGNYNYLYRLFRKSN | 445 | 0.71 |
| 14 | TGCVIAWNSNNLDSKV | 430 | 0.71 |
| 15 | FVIRGDEVRQIAPGQT | 400 | 0.69 |
| 16 | VVLSFELLHAPATVCG | 511 | 0.66 |
| 17 | RKSNLKPFERDISTEI | 457 | 0.65 |
| 18 | PTKLNDLCFTNVYADS | 384 | 0.62 |
| **NTD (Average : 0.733)** | | | |
| 1 | SWMESEFRVYSSANNC | 151 | 0.86 |
| 2 | KSNIIRGWIFGTTLDS | 97 | 0.84 |
| 3 | VSGTNGTKRFDNPVLP | 70 | 0.82 |
| 4 | TNVVIKVCEFQFCNDP | 124 | 0.80 |
| 5 | VYSSANNCTFEYVSQP | 159 | 0.79 |
| 6 | GTTLDSKTQSLLIVNN | 107 | 0.78 |
| 7 | CNDPFLGVYYHKNNKS | 136 | 0.73 |
| 8 | DGVYFASTEKSNIIRG | 88 | 0.72 |
| 9 | PFLMDLEGKQGNFKNL | 174 | 0.65 |
| 10 | PVLPFNDGVYFASTEK | 82 | 0.63 |
| 10 | TKRFDNPVLPFNDGVY | 76 | 0.63 |
| 11 | LIVNNATNVVIKVCEF | 118 | 0.55 |
