# Supplementary material for: Epitope-based chimeric peptide vaccine design against S, M and E proteins of SARS-CoV-2, the etiologic agent of COVID-19 pandemic: an in silico approach
Source: PeerJ. 2020 Jul 27;8:e9572. doi: 10.7717/peerj.9572 (PMC7394063; doi:10.7717/peerj.9572)
Supplement: Table S5 [file peerj-08-9572-s007.docx]

**Table S5:** IFN-γ inducing epitopes predicted by IFNepitope program

| **Serial No.** | **Start** | **Sequence** | **Method** | **Result** | **Score** |
| --- | --- | --- | --- | --- | --- |
| **NTD (average score :0.312)** | | | | | |
| 1 | 89 | VYFASTEKSNIIRGW | SVM | POSITIVE | 0.810945 |
| 2 | 90 | YFASTEKSNIIRGWI | SVM | POSITIVE | 0.78967 |
| 3 | 91 | FASTEKSNIIRGWIF | SVM | POSITIVE | 0.642475 |
| 4 | 88 | GVYFASTEKSNIIRG | SVM | POSITIVE | 0.637418 |
| 5 | 92 | ASTEKSNIIRGWIFG | SVM | POSITIVE | 0.502773 |
| 6 | 93 | STEKSNIIRGWIFGT | SVM | POSITIVE | 0.502331 |
| 7 | 94 | TEKSNIIRGWIFGTT | SVM | POSITIVE | 0.481011 |
| 8 | 137 | DPFLGVYYHKNNKSW | SVM | POSITIVE | 0.4484 |
| 9 | 167 | FEYVSQPFLMDLEGK | SVM | POSITIVE | 0.413322 |
| 10 | 95 | EKSNIIRGWIFGTTL | SVM | POSITIVE | 0.407048 |
| 11 | 132 | FQFCNDPFLGVYYHK | SVM | POSITIVE | 0.35935 |
| 12 | 131 | EFQFCNDPFLGVYYH | SVM | POSITIVE | 0.338283 |
| 13 | 87 | DGVYFASTEKSNIIR | SVM | POSITIVE | 0.305856 |
| 14 | 138 | PFLGVYYHKNNKSWM | SVM | POSITIVE | 0.301977 |
| 15 | 171 | SQPFLMDLEGKQGNF | SVM | POSITIVE | 0.2747 |
| 16 | 139 | FLGVYYHKNNKSWME | SVM | POSITIVE | 0.264761 |
| 17 | 136 | NDPFLGVYYHKNNKS | SVM | POSITIVE | 0.254982 |
| 18 | 168 | EYVSQPFLMDLEGKQ | SVM | POSITIVE | 0.25338 |
| 19 | 134 | FCNDPFLGVYYHKNN | SVM | POSITIVE | 0.239539 |
| 20 | 97 | SNIIRGWIFGTTLDS | SVM | POSITIVE | 0.226778 |
| 21 | 169 | YVSQPFLMDLEGKQG | SVM | POSITIVE | 0.210321 |
| 22 | 170 | VSQPFLMDLEGKQGN | SVM | POSITIVE | 0.198977 |
| 23 | 140 | LGVYYHKNNKSWMES | SVM | POSITIVE | 0.19694 |
| 24 | 56 | PFFSNVTWFHAIHVS | SVM | POSITIVE | 0.171456 |
| 25 | 133 | QFCNDPFLGVYYHKN | SVM | POSITIVE | 0.169759 |
| 26 | 166 | TFEYVSQPFLMDLEG | SVM | POSITIVE | 0.15245 |
| 27 | 141 | GVYYHKNNKSWMESE | SVM | POSITIVE | 0.150871 |
| 28 | 96 | KSNIIRGWIFGTTLD | SVM | POSITIVE | 0.10791 |
| 29 | 98 | NIIRGWIFGTTLDSK | SVM | POSITIVE | 0.10336 |
| 30 | 135 | CNDPFLGVYYHKNNK | SVM | POSITIVE | 0.098891 |
| 31 | 126 | VIKVCEFQFCNDPFL | SVM | POSITIVE | 0.094081 |
| 32 | 84 | PFNDGVYFASTEKSN | SVM | POSITIVE | 0.087175 |
| 33 | 172 | QPFLMDLEGKQGNFK | SVM | POSITIVE | 0.085353 |
| **RBD (Average score :0.255)** | | | | | |
| 1 | 462 | PFERDISTEIYQAGS | SVM | POSITIVE | 0.624815 |
| 2 | 463 | FERDISTEIYQAGST | SVM | POSITIVE | 0.572874 |
| 3 | 507 | YRVVVLSFELLHAPA | SVM | POSITIVE | 0.571759 |
| 4 | 460 | LKPFERDISTEIYQA | SVM | POSITIVE | 0.553997 |
| 5 | 345 | RFASVYAWNRKRISN | SVM | POSITIVE | 0.531663 |
| 6 | 465 | RDISTEIYQAGSTPC | SVM | POSITIVE | 0.525355 |
| 7 | 346 | FASVYAWNRKRISNC | SVM | POSITIVE | 0.522057 |
| 8 | 344 | TRFASVYAWNRKRIS | SVM | POSITIVE | 0.521612 |
| 9 | 506 | PYRVVVLSFELLHAP | SVM | POSITIVE | 0.520905 |
| 10 | 461 | KPFERDISTEIYQAG | SVM | POSITIVE | 0.514967 |
| 11 | 503 | GYQPYRVVVLSFELL | SVM | POSITIVE | 0.500094 |
| 12 | 464 | ERDISTEIYQAGSTP | SVM | POSITIVE | 0.490169 |
| 13 | 466 | DISTEIYQAGSTPCN | SVM | POSITIVE | 0.480013 |
| 14 | 347 | ASVYAWNRKRISNCV | SVM | POSITIVE | 0.474759 |
| 15 | 349 | VYAWNRKRISNCVAD | SVM | POSITIVE | 0.438636 |
| 16 | 502 | VGYQPYRVVVLSFEL | SVM | POSITIVE | 0.394901 |
| 17 | 389 | LCFTNVYADSFVIRG | SVM | POSITIVE | 0.366524 |
| 18 | 350 | YAWNRKRISNCVADY | SVM | POSITIVE | 0.35601 |
| 19 | 348 | SVYAWNRKRISNCVA | SVM | POSITIVE | 0.350959 |
| 20 | 505 | QPYRVVVLSFELLHA | SVM | POSITIVE | 0.345882 |
| 21 | 508 | RVVVLSFELLHAPAT | SVM | POSITIVE | 0.324276 |
| 22 | 504 | YQPYRVVVLSFELLH | SVM | POSITIVE | 0.318673 |
| 23 | 388 | DLCFTNVYADSFVIR | SVM | POSITIVE | 0.282876 |
| 24 | 467 | ISTEIYQAGSTPCNG | SVM | POSITIVE | 0.256374 |
| 25 | 343 | ATRFASVYAWNRKRI | SVM | POSITIVE | 0.243891 |
| 26 | 406 | VRQIAPGQTGKIADY | SVM | POSITIVE | 0.238696 |
| 27 | 470 | EIYQAGSTPCNGVEG | SVM | POSITIVE | 0.233029 |
| 28 | 509 | VVVLSFELLHAPATV | SVM | POSITIVE | 0.229693 |
| 29 | 408 | QIAPGQTGKIADYNY | SVM | POSITIVE | 0.22687 |
| 30 | 468 | STEIYQAGSTPCNGV | SVM | POSITIVE | 0.193904 |
| 31 | 510 | VVLSFELLHAPATVC | SVM | POSITIVE | 0.176479 |
| 32 | 342 | NATRFASVYAWNRKR | SVM | POSITIVE | 0.162741 |
| 33 | 354 | RKRISNCVADYSVLY | SVM | POSITIVE | 0.152176 |
| 34 | 407 | RQIAPGQTGKIADYN | SVM | POSITIVE | 0.143402 |
| 35 | 501 | GVGYQPYRVVVLSFE | SVM | POSITIVE | 0.12467 |
| 36 | 353 | NRKRISNCVADYSVL | SVM | POSITIVE | 0.120148 |
| 37 | 443 | KVGGNYNYLYRLFRK | SVM | POSITIVE | 0.114659 |
| 38 | 390 | CFTNVYADSFVIRGD | SVM | POSITIVE | 0.112158 |
| 39 | 351 | AWNRKRISNCVADYS | SVM | POSITIVE | 0.107934 |
| 40 | 365 | SVLYNSASFSTFKCY | SVM | POSITIVE | 0.106128 |
| 41 | 366 | VLYNSASFSTFKCYG | SVM | POSITIVE | 0.088337 |
| 42 | 492 | QSYGFQPTNGVGYQP | SVM | POSITIVE | 0.076122 |
| 43 | 444 | VGGNYNYLYRLFRKS | SVM | POSITIVE | 0.076019 |
| 44 | 409 | IAPGQTGKIADYNYK | SVM | POSITIVE | 0.068857 |
| 45 | 336 | PFGEVFNATRFASVY | SVM | POSITIVE | 0.064041 |
| 46 | 445 | GGNYNYLYRLFRKSN | SVM | POSITIVE | 0.06335 |
| 47 | 340 | VFNATRFASVYAWNR | SVM | POSITIVE | 0.05905 |
| 48 | 339 | GEVFNATRFASVYAW | SVM | POSITIVE | 0.058894 |
| 49 | 340 | EVFNATRFASVYAWN | SVM | POSITIVE | 0.043785 |
| 50 | 391 | FTNVYADSFVIRGDE | SVM | POSITIVE | 0.043701 |
| 51 | 459 | NLKPFERDISTEIYQ | SVM | POSITIVE | 0.030851 |
| 52 | 469 | TEIYQAGSTPCNGVE | SVM | POSITIVE | 0.024497 |
| 53 | 355 | KRISNCVADYSVLYN | SVM | POSITIVE | 0.016069 |
| 54 | 483 | EGFNCYFPLQSYGFQ | SVM | POSITIVE | 0.011051 |
| 55 | 511 | VLSFELLHAPATVCG | SVM | POSITIVE | 0.009809 |
| 56 | 357 | ISNCVADYSVLYNSA | SVM | POSITIVE | 6.87E-05 |
| MBE POSITIVE 0.97925313 | | | | | |
