# Supplementary figures and images for: Epitope-based chimeric peptide vaccine design against S, M and E proteins of SARS-CoV-2, the etiologic agent of COVID-19 pandemic: an in silico approach
Source: PeerJ. 2020 Jul 27;8:e9572. doi: 10.7717/peerj.9572 (PMC7394063; doi:10.7717/peerj.9572)

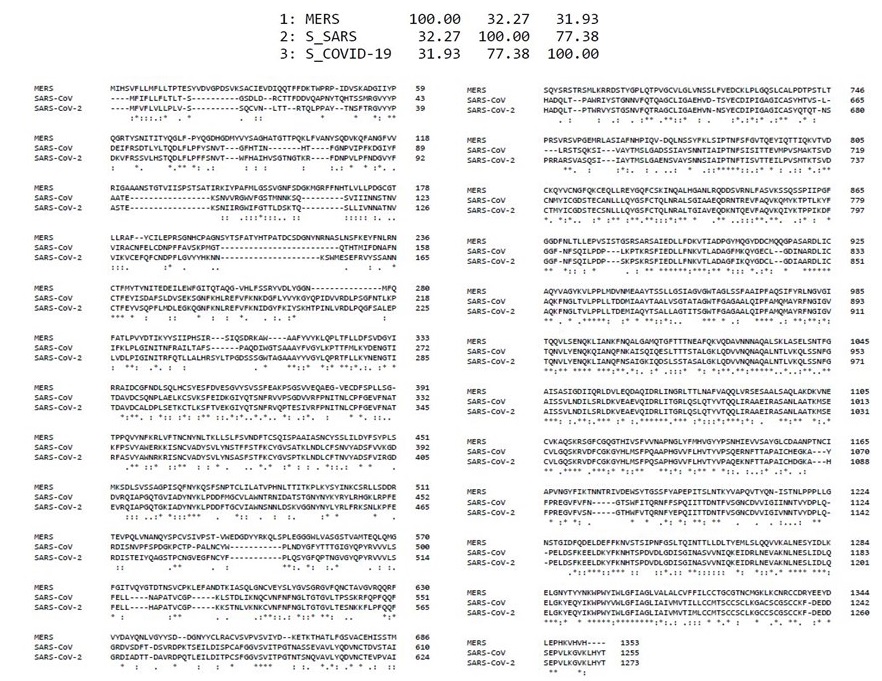

Supplement: Figure S1 — Using ClustalW multiple sequence alignment tool (version 1.2.4) we found that the S protein of SARS-CoV-2 shares 77.38% and 31.93% sequence identity with the S proteins of the SARS-CoV and MERS-CoV, respectively. [file peerj-08-9572-s008.jpg]

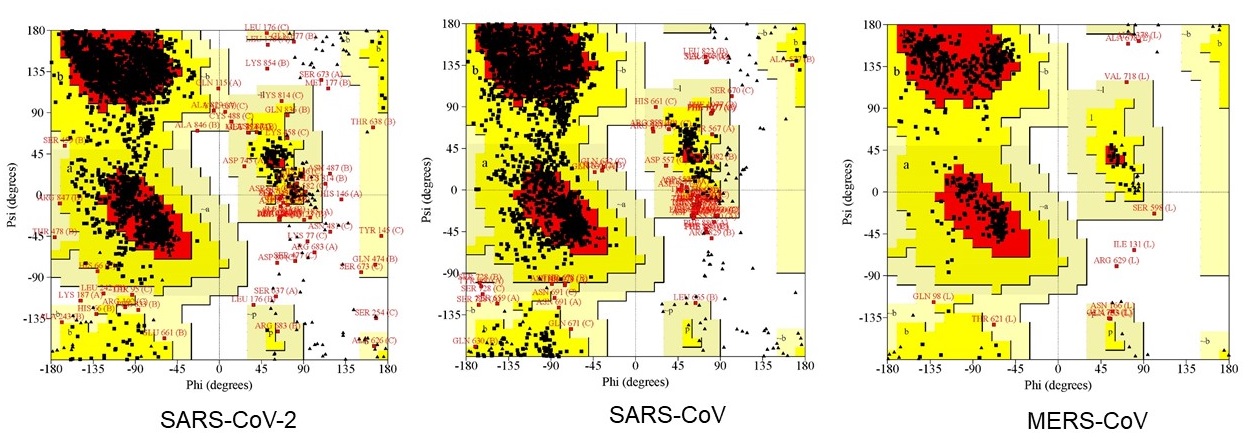

Supplement: Figure S2 — The three plots (a), (b) and (c) respectively illustrates the spike (S) proteins of SARS-CoV-2, SARS-CoV and MERS-CoV of Ramachandran outputs using PROCHECK web server. Most favored regions in the plots of are shown in red, additional allowed regions are shown in yellow, generously allowed regions are shown light brown, and disallowed regions are shown in white. After refinement, in SARS-CoV-2 S protein 85.1% and 12.7% amino acid residues were found in favored and allowed regions, respectively. The SARS-CoV S protein however had 78.4% and10.2% residues in favored and allowed regions, respectively, and 88.1% and 19.6% residues belonged to favored and allowed regions, respectively in MERS-CoV S protein. [file peerj-08-9572-s009.jpg]

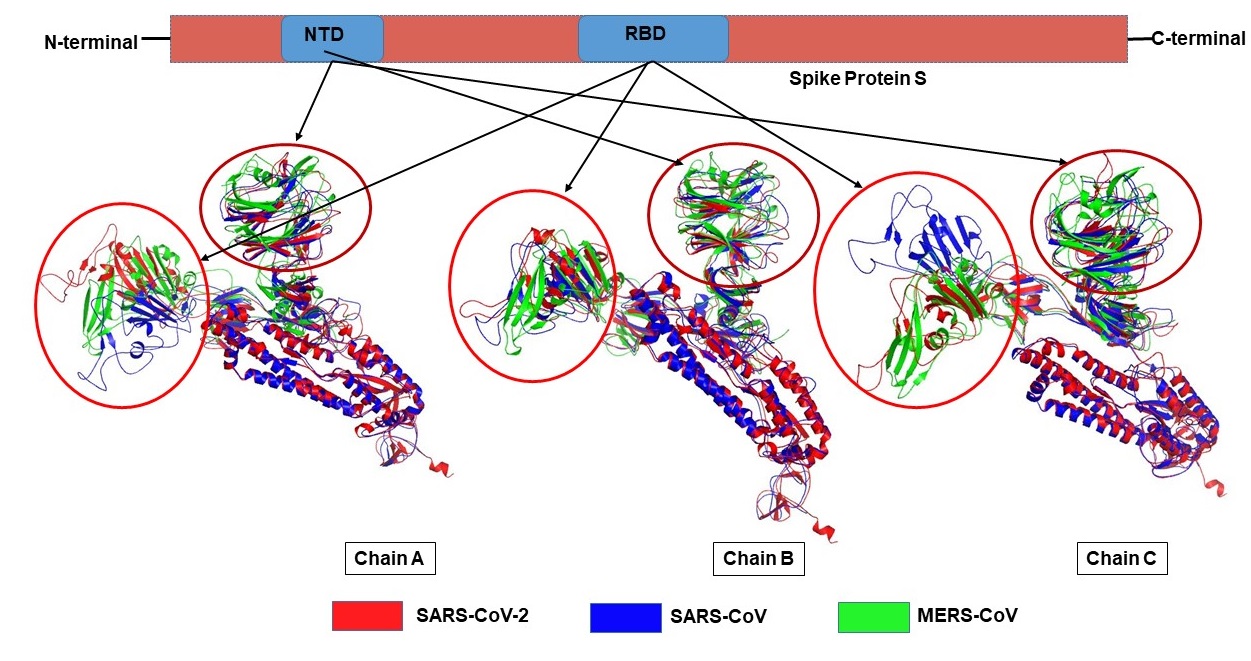

Supplement: Figure S3 — The S proteins of the SARS-CoV-2, SARS-CoV and MERS-CoV are trimeric conformation consisting of three homologous chains named as chain A, B and C . Structural alignment of these three chains using PyMOL revealed high degree of structural divergences in the N-terminal domains (NTDs) and receptor binding domains (RBDs) of the chains A and C compared to that of chain B. [file peerj-08-9572-s010.jpg]

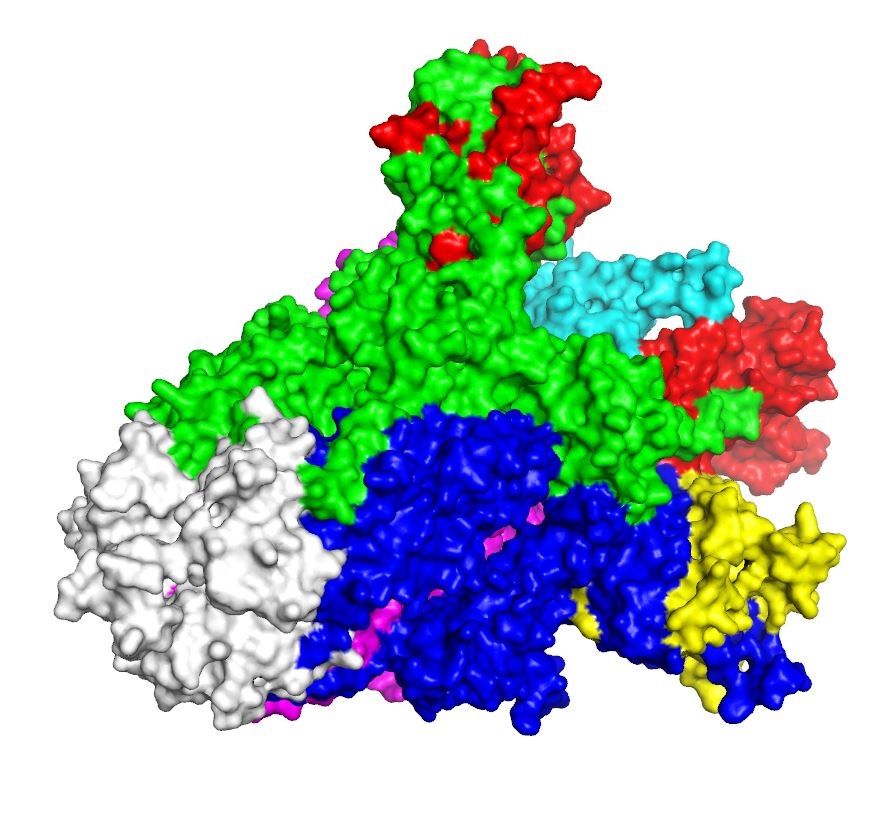

Supplement: Figure S4 — The red, cyan, and yellow colored regions represent the potential antigenic domains predicted by the IEDB analysis resource ElliPro analysis whereas the gray colored region represents the transmembrane domain of S protein. [file peerj-08-9572-s011.jpg]

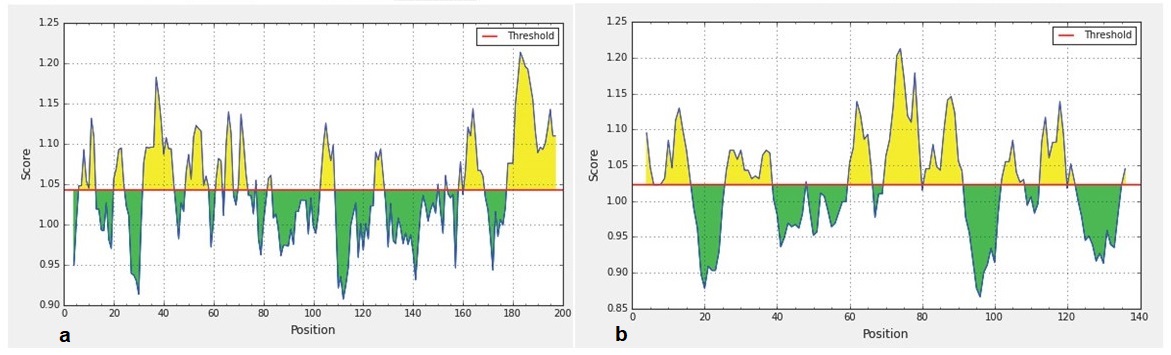

Supplement: Figure S5 — Yellow areas above threshold (red line) are proposed to be a part of B cell epitopes in (a) RBD and (b) NTD regions of S protein of the SARS-CoV-2. [file peerj-08-9572-s012.jpg]

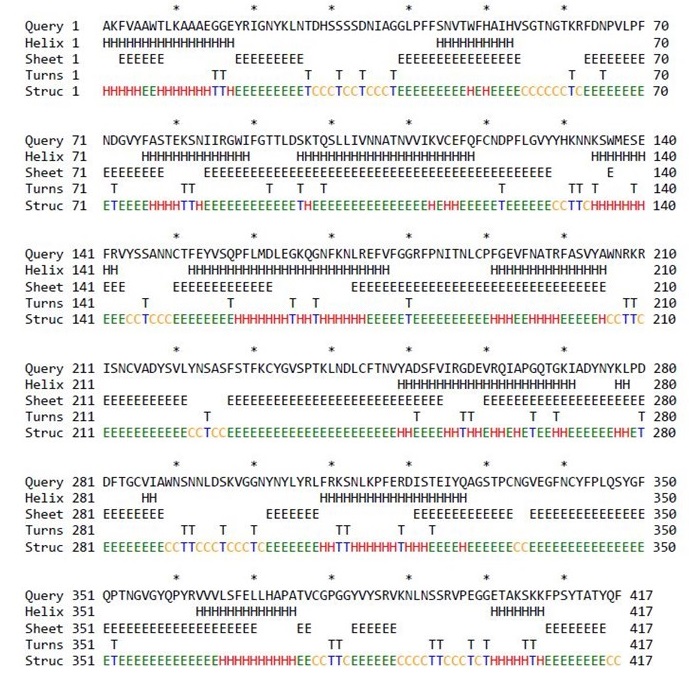

Supplement: Figure S6 [file peerj-08-9572-s013.jpg]

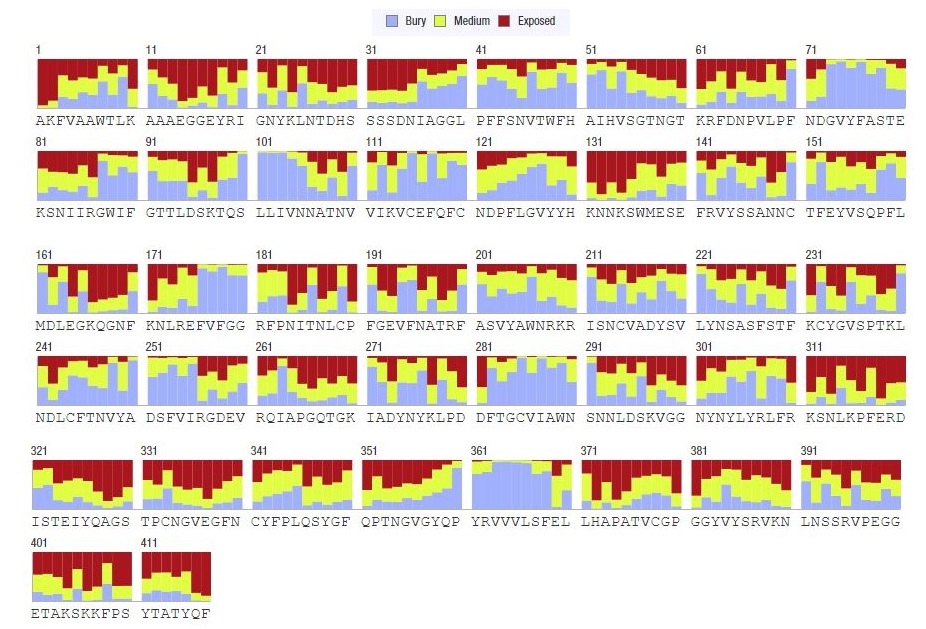

Supplement: Figure S7 [file peerj-08-9572-s014.jpg]

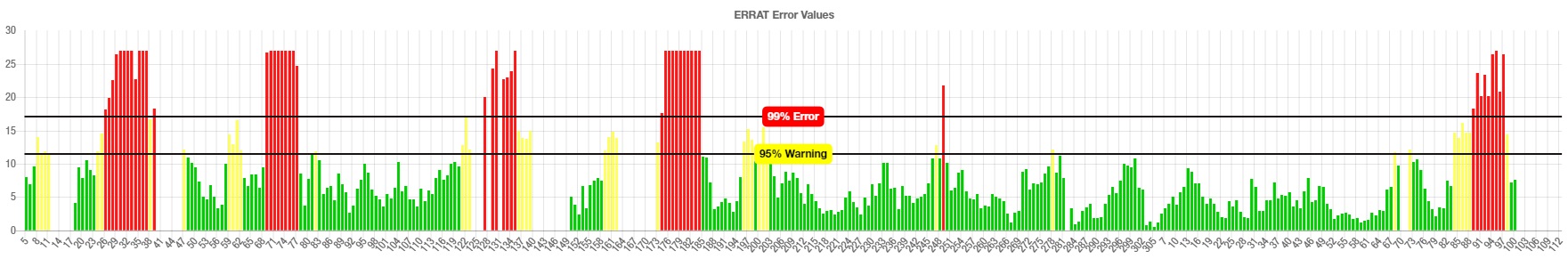

Supplement: Figure S8 [file peerj-08-9572-s015.jpg]

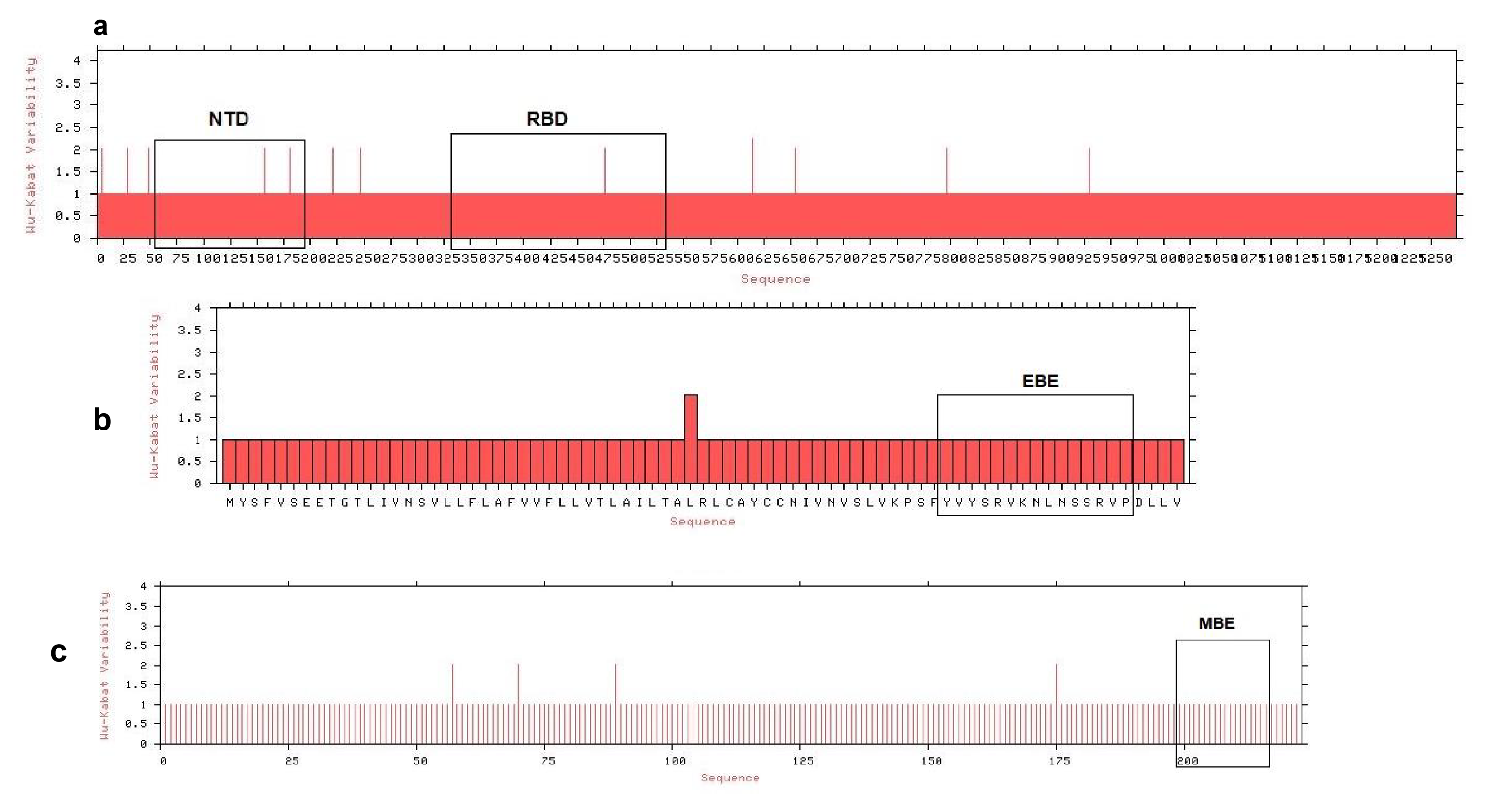

Supplement: Figure S9 — Variability plot of (a) spike (S) glycoprotein (b) membrane (M) protein and (c) envelope (E) protein. [file peerj-08-9572-s016.png]
